# Gut Malrotation in a Human Cadaver: Exploration Into the Prevention and Screening of Undiagnosed Malrotation in Adults

**DOI:** 10.7759/cureus.62318

**Published:** 2024-06-13

**Authors:** Madison Dominy, Mandeville Sofer, Meredith Slaby, Lindsay Slemmons, Nathan Smith, David Kashmer, Daniel Cawley

**Affiliations:** 1 Department of Biomedical Research and Affairs, Edward Via College of Osteopathic Medicine, Auburn, USA; 2 Department of Surgery and Simulation, Edward Via College of Osteopathic Medicine, Auburn, USA

**Keywords:** congenital, ladd's bands, adult, imaging, case report, intestinal malrotation

## Abstract

Malrotation is a congenital anomaly that results from the abnormal rotation of the gut during fetal development. Malrotation may be missed in early life and can present later with non-specific, chronic abdominal symptoms and decreased quality of life and in some cases can lead to serious bowel complications. Most adult cases are discovered incidentally on imaging or during surgery. An 82-year-old male cadaver was identified as having probable malrotation of the intestines. The performance of a previous surgical procedure could not be confirmed due to a lack of medical and surgical history. The cadaver dissection raised the question regarding the screening modalities used to reliably identify malrotations in infants and adults. Implementing a standardized reliable screening tool in infants or adults complaining of chronic abdominal pain could largely reduce the incidence of undiagnosed malrotation. Along with the development of a screening tool, increasing understanding of the clinical presentation of malrotation in adults could help identify undiagnosed cases earlier, which can reduce morbidity and mortality in these patients.

## Introduction

Intestinal malrotation is a congenital anomaly resulting from abnormal gut rotation during development, which leads to intestinal malposition and mesenteric fixation. Normally, between the sixth and 10th weeks of gestation, the midgut elongates and develops a series of loops that undergo physiological herniation followed by a return to the abdominal cavity. During this process, the midgut loops reposition within the abdominal cavity, with the duodenojejunal junction positioned to the left of the midline, the cecum positioned in the lower right quadrant, and the colon forming a "frame" around the more centrally located jejunum and ileum [1,2]. Historically, the intestinal rotation has been merely described as a 270° counterclockwise rotation around the superior mesenteric artery (SMA); however, the developmental positioning of the gastrointestinal tract is believed to be much more complex with many cellular pathways influencing differentiation and proper arrangement [1]. 

Intestinal malrotation most commonly presents in the first month of life with symptoms of acute bowel obstruction such as sudden bilious vomiting, colicky abdominal pain and distension, and failure to thrive. More severe symptoms, such as peritonitis and shock, can occur when the bowel becomes ischemic [3-5]. Malrotation diagnosed outside of infancy is often incidental, discovered on imaging during evaluation for other abdominal diseases, most commonly for concerns of small bowel volvulus [6-9]. Adults with a final diagnosis of malrotation will normally present with recurring non-specific abdominal pain, nausea, and vomiting. Moreover, some individuals with these presenting symptoms will not be diagnosed with malrotation until later despite having undergone a previous surgical procedure [10]. The risk of not diagnosing malrotation in infancy leaves the individual at a higher frequency of developing biliary atresia, chronic pancreatitis, and intermittent episodes of spontaneously resolving duodenal obstruction. However, some patients may never experience symptoms or complications. The current surgical treatment approach used in pediatric and adult populations was discovered by William E. Ladd in 1936 and consists of peritoneal bands (Ladd's bands) division, volvulus reduction if present, appendectomy, and functional repositioning of the intestines. Both open and laparoscopic Ladd's procedures are utilized in both populations. The open approach is currently considered the gold standard in infants, although the laparoscopic procedure is becoming more commonly utilized in both infant and adult populations [11-13]. 

Adult malrotation is believed to be underdiagnosed with estimates approaching up to 1% of the human population [14].  Here, we present a case of intestinal malrotation discovered during cadaveric dissection and discuss the need for a more standardized approach to imaging analysis. 

## Case presentation

**Figure 1:**
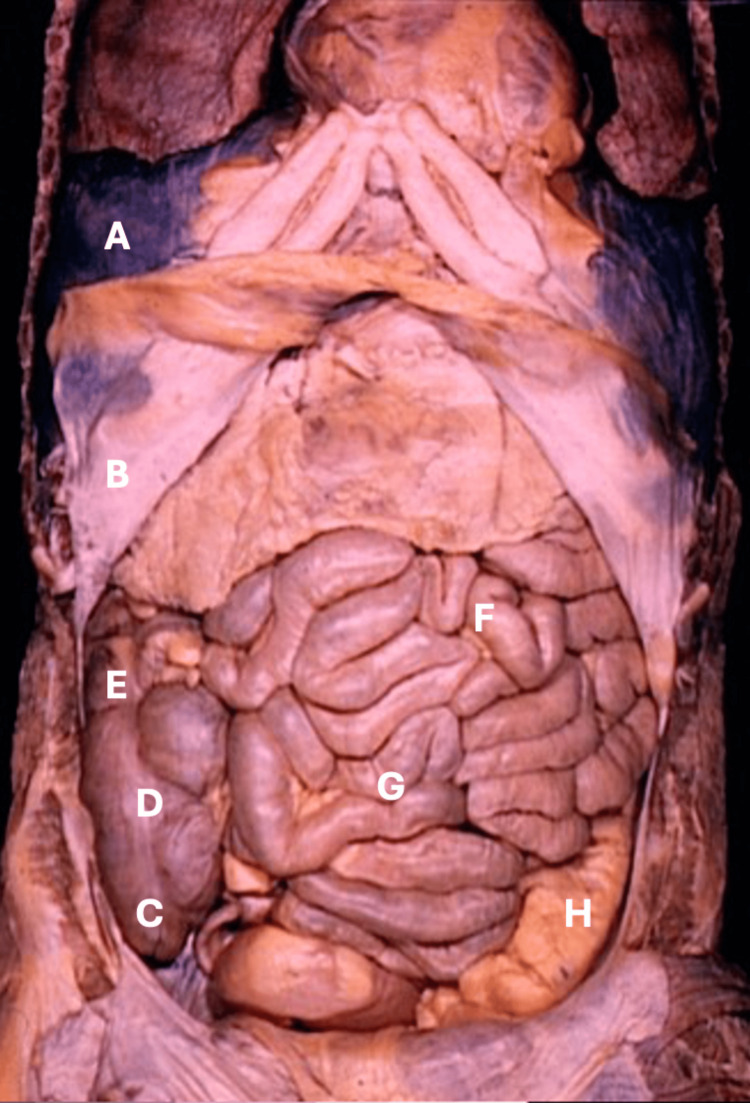
Normal intestinal positioning. (A) Diaphragm. (B) Costal margin. (C) Cecum. (D) Free taenia of the cecum. (E) Ascending colon. (F) Jejunum. (G) Ileum. (H) Sigmoid colon

**Figure 2:**
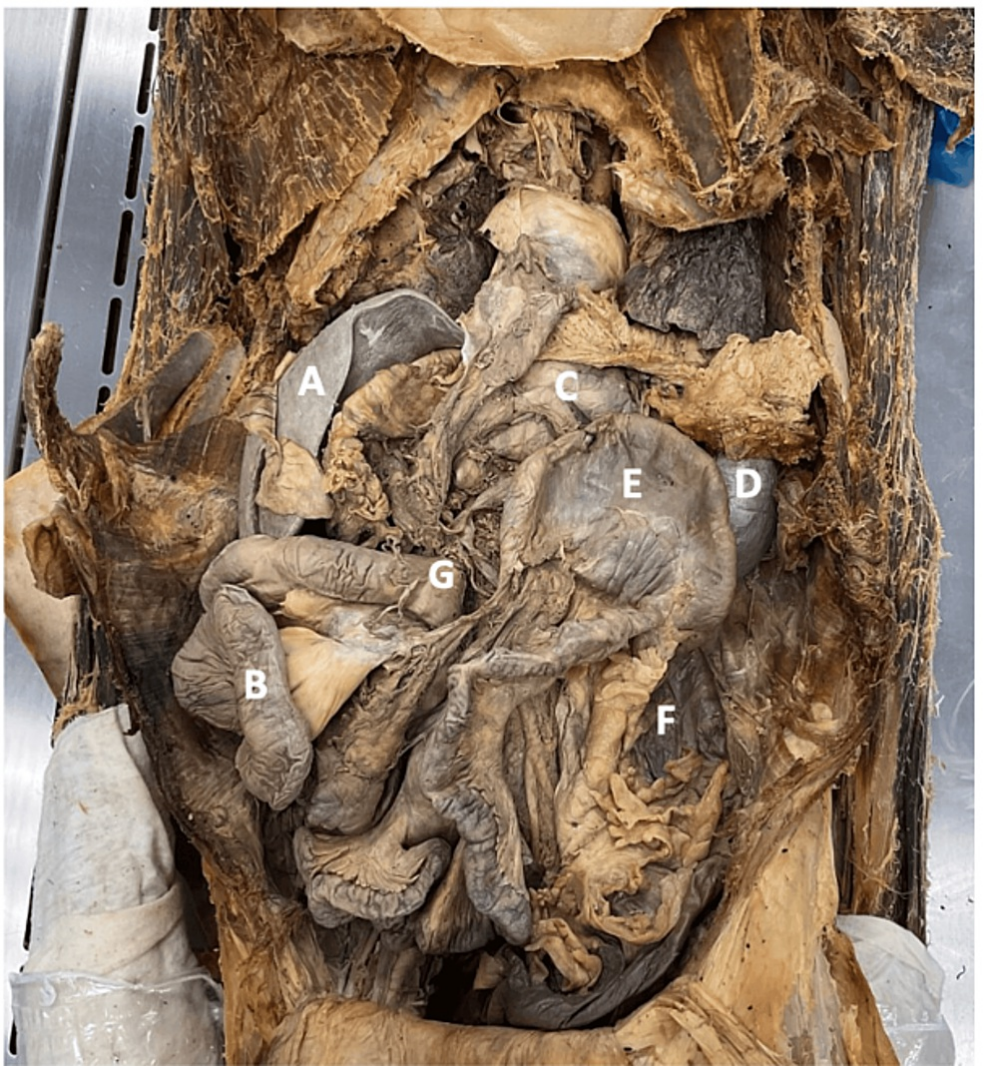
The small intestines are located on the right side of the abdominal cavity and the large intestine is located on the left. The duodenojejunal junction is positioned to the right of the midline as opposed to crossing to the left. This is a common criterion for malrotation. (A) Liver. (B) Small intestines. (C) Stomach. (D) Spleen. (E) Cecum. (F) Colon. (G) Duodenojejunal junction

A cadaveric dissection of an 82-year-old male cadaver in a first-year medical gross anatomy course revealed probable malrotation of the intestines. The skin and superficial fascia were removed by making an incision starting at the xiphoid process and proceeding laterally along the costal margin to the midaxillary line. The cut was extended inferiorly along the midaxillary line to a point about 3 cm below the anterior superior iliac spine and then continued inferomedially to the pubic symphysis. Following the removal of the skin and superficial fascia, the anterior abdominal wall was reflected by making an incision through all layers of the anterior abdominal wall, mirroring the skin incision lines; however, the cut was stopped above the inguinal ligament, 2 cm lateral to the deep inguinal ring. After reflecting the anterior abdominal wall inferiorly, the greater omentum was identified and cut away to allow more clear visualization of the abdominal content. Compared to normal anatomical positioning (Figure 1) [2], it was noted that the small and large intestines were not located in their normal anatomical position; instead, the small and large intestines were positioned predominantly on the right and left sides of the abdominal cavity, respectively. The duodenojejunal junction was not in its typical location, being positioned to the right of the midline as opposed to its normal position to the left of the midline (Figure 2).

**Figure 3:**
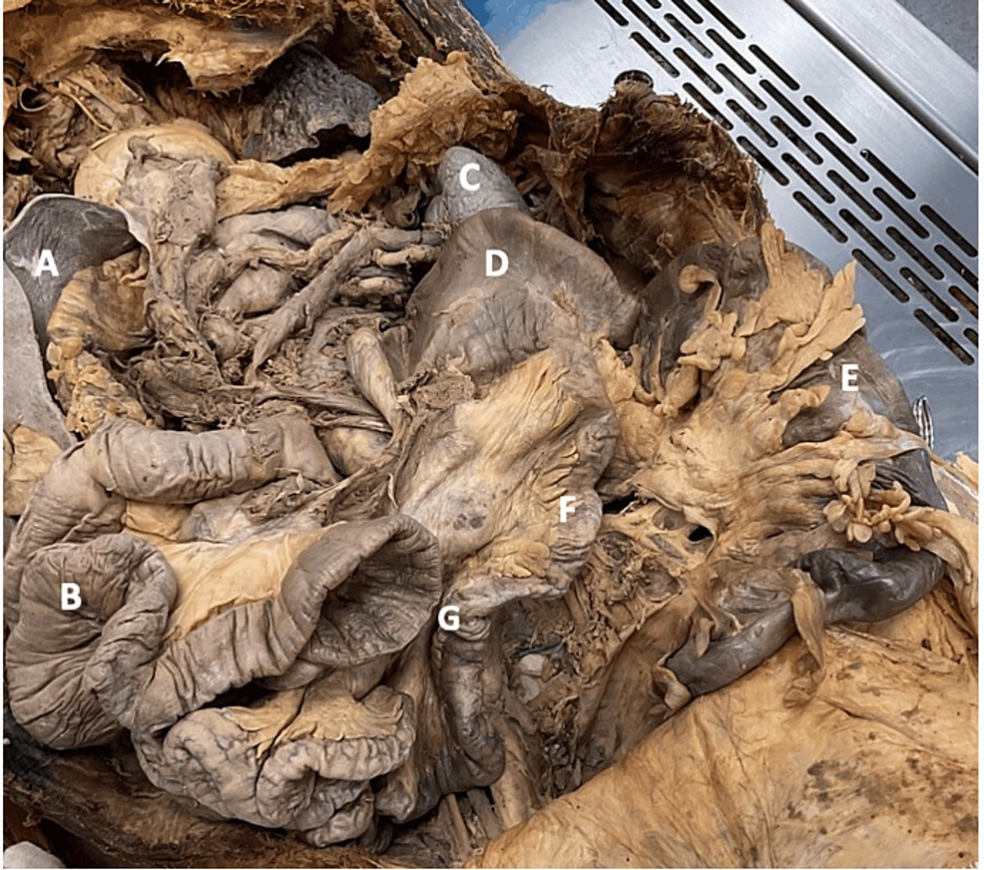
The large intestines consisted of a subsplenic cecum and an apparent shortened, left-sided colon that began at the splenorenal recess and terminated at the recto-sigmoidal junction. (A) Liver. (B) Jejunum. (C) Spleen. (D) Cecum. (E) Colon. (F) Ileum. (G) Duodenojejunal junction

The liver, gallbladder, and spleen were in their normal position, with the liver and gallbladder located primarily in the right upper quadrant and the spleen in the left upper quadrant. The base of the small intestine mesentery appeared narrowed with a smaller attachment area to the posterior abdominal wall. The large intestine consisted of a subsplenic cecum that terminated at the recto-sigmoidal junction (Figure 3).

**Figure 4:**
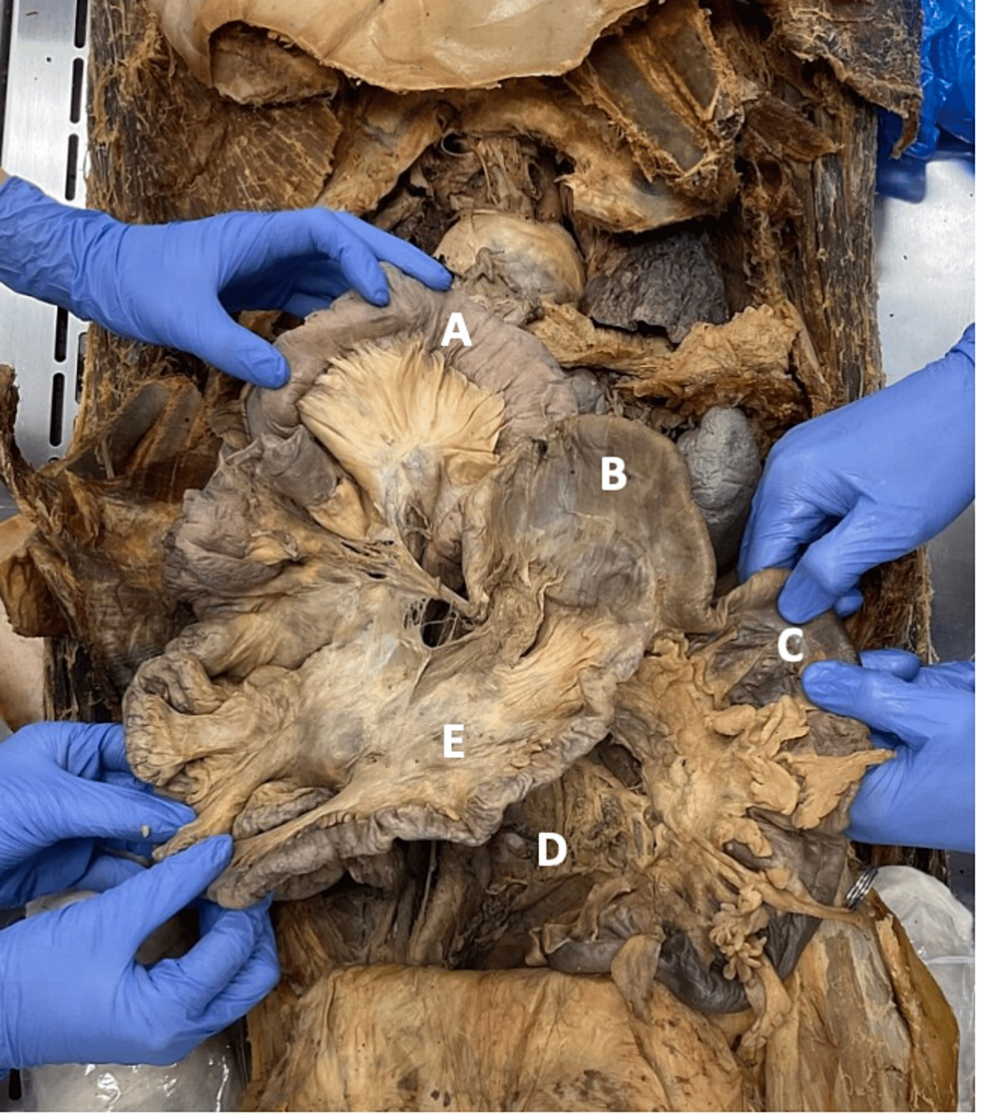
Absence of the appendix raises suspicion for a previous corrective surgery, a Ladd's procedure. Evidence of the procedure could not be confirmed definitively. (A) Jejunum. (B) Cecum. (C) Colon. (D) Recto-sigmoidal junction. (E) Ileum

Only a portion of the colon was fused to the posterior abdominal wall with the majority of the colon being free and mobile. Absence of the appendix raises suspicion for a previous corrective surgery, a Ladd's procedure, which typically involves an appendectomy (Figure 4).

Evidence of the procedure could not be confirmed definitively due to the lack of medical history, so the removal of the appendix could be due to a prior unrelated appendectomy or due to an undocumented Ladd's procedure. Furthermore, no other signs of additional abdominal pathology or prior abdominal surgeries were noted.

## Discussion

Here, we present a case of possible intestinal malrotation in a cadaver dissected during a medical school gross anatomy course. The positioning of the colon and small intestines to the left and right sides of the abdominal cavity, respectively, would be present in cases of intestinal nonrotation or individuals with malrotation who have undergone a Ladd's procedure. The presence of a right-sided duodenojejunal junction, narrowed small intestinal mesentery attachment, and the lack of an appendix provide evidence for the latter; however, this cannot be proven conclusively without a medical history. 

**Figure 5:**
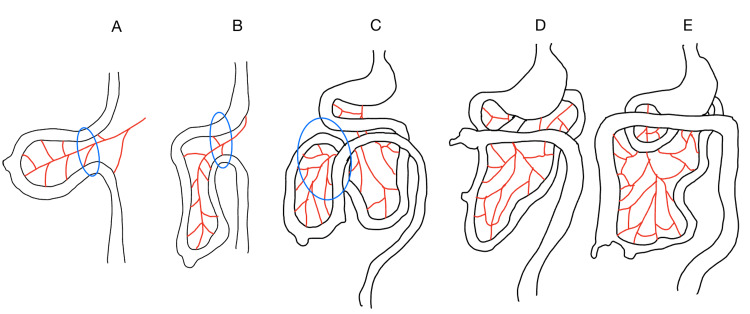
Normal rotation of the small and large intestines during embryologic development. This figure represents the normal rotation of the small and large intestines during embryologic development. The red lines represent the SMA and its branches, and the blue circle represents the ventral body wall. (A) 6 weeks gestation. (B) 8 weeks gestation. (C) 9 weeks gestation. (D) 11 weeks gestation. (E) 12 weeks gestation Figure drawn by author Mandeville Sofer based on [15]. SMA: superior mesenteric artery

Intestinal malrotation is a developmental abnormality that occurs due to the disruption of the normal rotation of the intestines during development (Figure 5).

**Figure 6:**
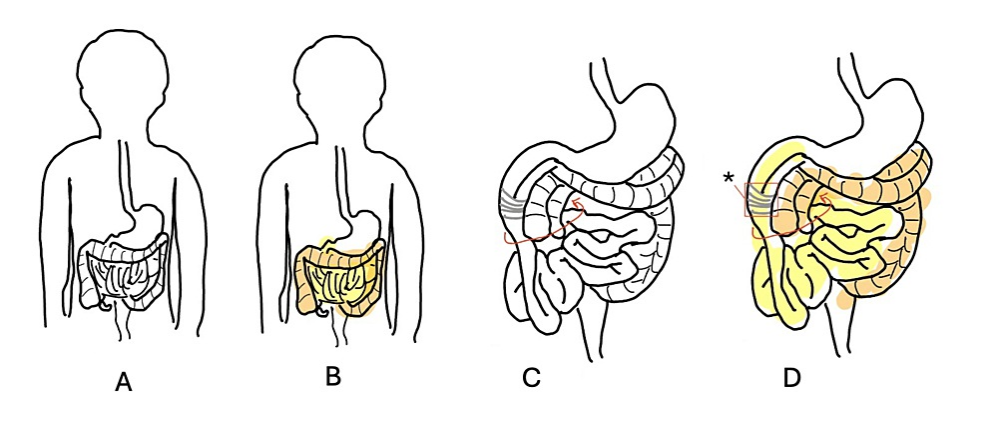
Positioning of intestinal anatomy in normal vs malrotation patients. (A) Normal. (B) Normal positioning with orange highlighting the large intestine and yellow highlighting the small intestine. (C) Malrotation without volvulus. (D) Malrotation without volvulus, with orange highlighting the large intestines and yellow highlighting small intestines and an asterisk identifying Ladd's bands Figure drawn by author Mandeville Sofer based on [19].

This leads to inappropriate positioning and fixation by Ladd's bands, which can cause an intestinal volvulus (Figure 6) leading to gastrointestinal obstruction, ischemia, and pain [16-18].

Complications of malrotation, and therefore diagnoses, are commonly delayed. In the United States, it is estimated that one in 500 live births will have some degree of malrotation with only about one in 6000 live births showing signs and symptoms of midgut malrotation [10,20]. In a retrospective study performed by Nehra and Goldstein [21], malrotation was newly diagnosed in 31% of infants and 21% of children aged 1-18 years, with the remaining 43% diagnosed in adulthood. Their study demonstrated that 118 out of 170 patients were older than one year, with 82 patients being older than 18 years of age. Malrotation is diagnosed less in adults, most likely due to the common presentation of non-specific, chronic abdominal symptoms such as intermittent abdominal pain, food intolerance, emesis, and diarrhea [6,7,21,22]. Chronic, non-specific abdominal symptoms overlap with many other conditions, resulting in a delay in proper diagnosis and treatment for months to years. In many cases, adult malrotation is asymptomatic and only discovered incidentally on imaging studies or laparotomy [7].  

The traditional "gold standard" diagnostic tool in pediatric malrotation is contrast upper gastrointestinal (UGI) imaging series demonstrating abnormal positioning of the duodenojejunal junction [23,24]. Other findings include a Z-shaped duodenum, if obstructing peritoneal bands are present, or a corkscrew-shaped duodenum, if volvulus is present; however, small intestine variations, such as a jejunal malposition, a redundant duodenum, or a mobile cecum, can increase the false-negative and false-positive rates or make diagnosis uncertain [23-25]. Plain radiography is often used initially in both the pediatric and adult populations and may identify possible signs of malrotation like intestinal obstruction. However, in the adult population, malrotation can be easily missed, with only the more severe cases of duodenal obstruction presenting with the "double-bubble sign" [23]. Therefore, the use of plain radiography is not useful, due to its inability to directly demonstrate actual malrotation. A meta-analysis by Nguyen et al. [26] of 17 studies consisting of over 2200 patients demonstrated excellent diagnostic accuracy of abdominal ultrasound when compared to other reference standards (computed tomography (CT), UGI, surgery, clinical follow-up) in individuals under the age of 21. When compared to UGI, ultrasound demonstrated a similar sensitivity, false-positive rate, and false-negative rate; however, ultrasound had a significantly higher specificity. A study by Taylor showed that CT has a sensitivity and specificity of 97.3% and 99%, respectively, in children; however, anatomical variation of the SMA can lower the accuracy to 76.8% [27].  

CT imaging is the gold standard in the diagnosis of adult malrotation, with one study reporting CT being diagnostic in 97.5% of cases [22]. The most common features of malrotation to present on CT imaging are abnormalities of the SMA and positional abnormalities of the small intestines and cecum [22]. Magnetic resonance imaging (MRI) has a poorer diagnostic yield and is not considered the gold standard in acute settings [20]. Findings, however, are similar to that of CT with the superior mesenteric vein (SMV) anterior and to the left of the SMA [28]. CT imaging of malrotation in adults typically presents with the findings of whirlpool and corkscrew signs or reversed relation of SMA and SMV [17]. These findings are highly specific for midgut malrotation; however, they are often identified only in severe symptomatic cases with volvulus. Asymptomatic, incidental findings on CT that demonstrates malrotation that does not warrant further evaluation can become problematic to the patient when this information is never fully explained or identified until severe symptoms present in the future. 

Because malrotation in adults is rare, many practitioners misidentify conventional radiographic imaging, which explains why CT scanning is more sensitive in findings of malrotation [7]. However, many findings of malrotation with CT alone can be difficult to identify and differentiate from other abdominal diagnoses in patients presenting with other comorbidities. The need for a uniform imaging protocol to rule out malrotation in the presence of complex imaging findings and non-specific abdominal symptoms could be beneficial in preventing more serious complications such as gangrene and bowel death in the future. Intestinal malrotation should be considered in the differential diagnosis in patients who present with unexplained, chronic recurrent abdominal pain and vomiting. A retrospective study by Chandra et al. [29] discovered that pancreatic head abnormalities and contour changes were frequently seen on CT scans of individuals with malrotation. A screening using CT colonography and incomplete optical colonoscopy has been proposed by Perez and Pickhardt [30]. They evaluated the prevalence and imaging findings, such as cross-sectional images of bowel malposition (e.g., proximal small bowel occupying the right abdomen) and an abnormal SMA-SMV relationship, in asymptomatic adults and found that intestinal malrotation was four times more likely to be missed in patients who were not given a CT colonography. Additionally, they found that many of these patients had some history of abdominal issues that had been attributed to other causes [30]. The different imaging modalities utilized in the diagnosis of pediatric and adult malrotation are presented in Table 1. Currently, there is no standard screening method to help diagnose potential malrotation in adults presenting with chronic abdominal symptoms. Increasing the understanding of the clinical presentation and imaging findings of adult malrotation could help identify undiagnosed cases earlier, possibly reducing the risk of developing future complications.

**Table 1:** Imaging findings in malrotation by modality. UGI: upper gastrointestinal; CT: computed tomography; MRI: magnetic resonance imaging; SMV: superior mesenteric vein; SMA: superior mesenteric artery

Imaging modality	Indication	Common findings	Additional comments
Plain radiography	Often used initially in pediatric and adult populations	May identify possible signs of malrotation (i.e., intestinal obstruction) [21]	Not very sensitive nor specific; does not specifically identify malrotation
Abdominal ultrasound		1) SMV to the left of the SMA (inversion of the SMA and SMV); 2) whirlpool sign for midgut volvulus, clockwise wrapping of the SMV and mesentery around the SMA [25]	Studies performed primarily in the pediatric population and not in adult populations
Contrast UGI imaging series	Traditional "gold standard" diagnostic tool in pediatric malrotation	Z-shaped duodenum if obstructing peritoneal band is present or corkscrew-shaped duodenum if volvulus is present [24,26]. Malrotation without volvulus shows abnormal positioning of the duodenojejunal junction [24,26]	
CT	Gold standard for diagnosis in adults	Abnormalities of the SMA, reversed relation of the SMA and SMV, whirlpool sign, and positional abnormalities of the small intestines and cecum [22,23]. Pancreatic head abnormalities and contour changes [29]	
CT colonography and incomplete optical colonoscopy		Cross-sectional imaging findings of bowel malposition (e.g., proximal small bowel occupying the right abdomen) are frequently evident as well as an abnormal SMA-SMV relationship in nearly 90% of cases [30]	Intestinal malrotation was four times more likely to be missed in patients who were not given a CT colonography. Of note, 10 of the adults with malrotation in the CT colonography screening cohort did not carry a pre-existing diagnosis, representing an undiagnosed prevalence of 0.1% or 1:1000 adults [30]
MRI		SMV anterior and to the left of SMA [28]	

## Conclusions

The cadaveric dissection of an 82-year-old male raised curiosity and suspicion of an intestinal nonrotation or an intestinal malrotation that underwent a previous Ladd's procedure. Practitioners could greatly benefit from the improvement of methods for earlier diagnosis, allowing them to further explore the rare possibility of malrotation being the cause of the chronic abdominal manifestations. Finding a uniform way of imaging and clearly identifying malrotation without exploratory surgery would greatly improve the quality of life of the patients, as this gap in medical diagnosis has yet to be fully addressed. The development of an imaging tool could allow for earlier intervention and the prevention of severe intestinal injury in the affected adult population. Instead of a diagnosis of exclusion, intestinal malrotation should be considered in the differential diagnosis in adults presenting with unexplainable abdominal symptoms. Newer imaging analyses, for instance, CT colonography, have been published and should be considered as part of a standardized imaging workflow used to diagnose intestinal malrotation when further imaging is needed. These considerations, along with a more widespread understanding among healthcare providers, could improve earlier identification of intestinal malrotation in adults. 
